# Unexpected species diversity of Malagasy primates (*Lepilemur *spp.) in the same biogeographical zone: a morphological and molecular approach with the description of two new species

**DOI:** 10.1186/1471-2148-7-83

**Published:** 2007-05-31

**Authors:** Mathias Craul, Elke Zimmermann, Solofonirina Rasoloharijaona, Blanchard Randrianambinina, Ute Radespiel

**Affiliations:** 1 Institute of Zoology, University of Veterinary Medicine Hannover, Buenteweg 17, 30559 Hannover, Germany; 2 Faculté des Sciences, Université d'Antananarivo, Antananarivo 101, Madagascar

## Abstract

**Background:**

The lemurs of Madagascar provide an excellent mammalian radiation to explore mechanisms and processes favouring species diversity and evolution. Species diversity, in particular of nocturnal species, increased considerably during the last decade. However, the factors contributing to this high diversity are not well understood. We tested predictions derived from two existing biogeographic models by exploring the genetic and morphological divergence among populations of a widely distributed lemur genus, the sportive lemur (*Lepilemur *ssp.) along a 560 km long transect from western to northern Madagascar.

**Results:**

By using the phylogenetic analyses of mtDNA sequence data, molecular diagnostic sites and phenotypic morphometric traits, we uncovered two previously undetected species whose distributions contradict the two existing biogeographic models. Brief species descriptions are provided and a new biogeographic model is proposed (the ”large river model“).

**Conclusion:**

According to the ”large river model“, large rivers in north and northwestern Madagascar acted as geographical barriers for gene flow and facilitated speciation events on a much smaller spatial scale than previously thought. Thereby, this study does not only show that species diversity in nocturnal Malagasy primates is continuously underestimated but aims to emphasize the need for conservation actions if those species with small ranges shall not face extinction in the near future.

## Background

Malagasy lemurs constitute one of six major radiations of extant primates [1]. Lemurs show a remarkable species diversity, both numerically and in terms of adaptations making them an excellent mammalian radiation to explore mechanisms and processes underlying speciation and evolution. During the last decade, species diversity in lemurs increased from 33 to currently 74 [2,3]. In relation to the small surface area of Madagascar, diversity of species within this primate radiation is quite high. Individual lemur species tend to have small geographic ranges in comparison to other primates. Because of such limited geographic ranges and the high rate of deforestation, the need for conservation action including genetic monitoring and effective management policies is particularly urgent [2,4]. Two major models have been proposed to explain diversity of Malagasy mammals.

**Figure 1:**
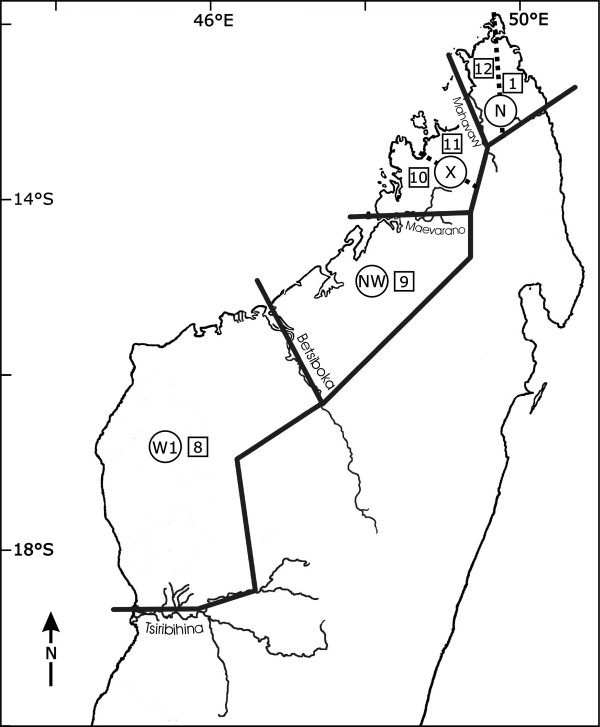
Zonation of northwestern Madagascar described by Martin (1972), zones marked with letters, and by Wilmé et al. (2006), zones marked with numbers.

The "Martin model" divided northern and northwestern Madagascar into four biogeographical zones (circles in Fig. 1) [5,6]. The western zone (W1) covers the area between the two major rivers Tsiribihina and Betsiboka. The northwestern zone 1 covers the area between the two major rivers Betsiboka and Maevarano (NW), the northwestern zone 2 the area between the rivers Maevarano and Mahavavy (X). The northern zone (N) covers the area between the rivers Mahavavy and Fanambana. These riverine barriers were hypothesized to form geographical boundaries to gene flow and consequently favour allopatric speciation. This model of speciation within Madagascar was refined [6] and it was shown that it is compatible with a reconstruction of speciation within the families Lemuridae, Cheirogaleidae and Indridae [7].

The recent "Wilmé model" explained the process of explosive speciation on the island using a mechanistic model [8]. Madagascar's rivers and associated watersheds with sources at relatively low elevations suggested to be zones of isolation that led to the evolution of locally endemic taxa, whereas those at higher elevations were proposed to have functioned as zones of retreat and dispersion and contain a lower level of microendemism. Wilmé et al. (2006) divided northern and northwestern Madagascar into six centres of endemism (squares in Fig. 1). The western zone (zone 8) covers the area between the two major rivers Tsiribihina and Betsiboka, corresponding to Martin's W1. One large northwestern zone, zone 9, corresponded to Martin's NW. Two smaller northwestern zones, zone 10, between the two rivers Maevarano and Sambirano, and zone 11 between the two rivers Sambirano and Mahavavy were suggested. In addition, two northern zones, one (zone 12), between the river Mahavavy and the continental divide between eastern and western draining watersheds, and another (zone 1), between the continental divide and the river Bemarivo, divided the N-zone of Martin into two partitions.

The geographical settings in northwestern and northern Madagascar are perfect to test if allopatric speciation of a widely distributed lemur genus follows one of the models. Each model predicts a different minimum number of species in this region and divergent distributions. Whereas the "Martin model" predicts four species, the "Wilmé model" proposes six species.

Sportive lemurs (*Lepilemur *spp.) are an excellent lemur group to test these two models of mammalian distribution in Madagascar, because they occur in almost all forested regions on the island. They are cat-sized vertical clingers and leapers with powerful hind legs. They are nocturnal and totally arboreal. They live in dispersed pairs and have an elaborated vocal repertoire [9-11]. Because differences in pelage colouration and other external characteristics between species are inconspicous, their early classification [12,13] based on morphological features was disputed until comprehensive cytogenetic approaches and molecular studies allowed the recognition of twelve species [11,14-18].

The aim of this study is to test the predictions from the models with the largest available genetic and morphological data set of a larger-sized lemur. We sequenced three mitochondrial genes of particular diagnostic importance for phylogeography (D-loop, Cytochrome B and NADH-dehydrogenase subunit 4) of individuals captured in 14 different localities that covered a 560 km transect and the area between eight large rivers (Inter-River-Systems, IRS) from western to northern Madagascar. In addition, morphometric data were analysed in order to explore, to which extent genetic differentiation coincides with morphological diversification. As in similar studies [e.g. [19,20]] we favour the phylogenetic species concept [21,22], where fixed molecular differences among parapatric populations indicate the existence of species barriers.

## Results

### Phylogenetic relationships

The 48 sequences available for the D-loop (43 own sequences + five reference sequences), after having cut out the hypervariable part, varied from 388 to 390 bp in length. 128 characters were constant, 201 variable characters are parsimony-uninformative and 66 were parsimony-informative. There were 17 different haplotypes. The 72 sequences available for the partial cytochrome B (43 own sequences + 29 reference sequences) were 352 bp long, with no indels. 211 characters were constant, 17 variable characters are parsimony-uninformative and 124 were parsimony-informative. There were 32 different haplotypes. The 50 sequences available for the partial ND4 (43 own sequences + seven reference sequences) varied from 630 to 631 bp in length. 408 characters were constant, 72 variable characters were parsimony-uninformative and 153 were parsimony-informative characters. There were 19 different haplotypes. Table 1 shows the best-fit models for the three loci selected by the hierarchical likelihood ratio test (hLRT) implemented in Modeltest 3.5.mac. Based on the single-gene-trees, derived for the new and reference sequences, the samples in this study could be classified as follows: The individuals found in IRS 0 clustered with *L. aeeclis*, the individuals found in IRS I with *L. edwardsi*, the individuals found in IRS IV with *L. sahamalazensis*, the individuals found in IRS V and VI with *L. dorsalis*, the individuals found in IRS VII with *L. ankaranensis*, the individuals from Kirindy with *L. ruficaudatus *and the individuals from Mantadia with *L. mustelinus*. The individuals from IRS II and III did not cluster with any of the reference sequences. No sampled individual clustered with the reference sequences of *L. leucopus*, *L. microdon*, *L. randrianasoli *or *L. septentrionalis*.

**Table 1:** Best-fit mutation model for the three mitochondrial loci and the concatenated sequence selected by the hierarchical likelihood ratio test (hLRT) implemented in Modeltest 3.5.mac.

Locus	Method	Model	Base	Nst	Alpha	Pinvar	TRatio
D-loop	ML, NJ	HKY+G	0.3066 0.2151 0.1828 0.2955	2	0.1752	0	3.2075
Cyt B	ML, NJ	HKY+I+G	0.3092 0.3229 0.1219 0.2460	2	3.5808	0.5469	12.5738
ND4	ML, NJ	HKY+G	0.3372 0.2697 0.1127 0.2804	2	0.2736	0	8.1268
concatenated	ML, NJ	HKY+I+G	0.3124 0.2661 0.1377 0.2838	2	0.8801	0.3833	6.1953

Nst: number of substitution types

Pinvar: assumed proportion of invariable sites

Alpha: shape parameter

TRatio: transition/transversion ratio

**Figure 2:**
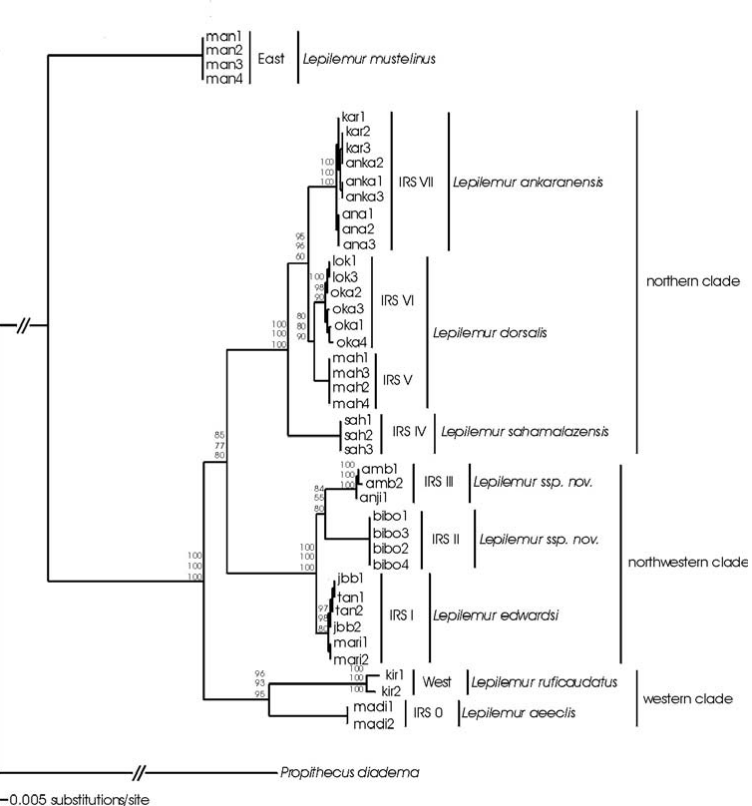
Neighbour-Joining tree based on the concatenated sequences of the three loci. The branch lengths indicate the number of substitutions, the numbers at the nodes indicate bootstrap values for internal branches (top: NJ, middle: MP, bottom: ML).

In order to reconstruct the phylogenetic relationships within the genus *Lepilemur*, we combined these three loci to one concatenated sequence, 1380 bp in length. 768 characters were constant, 333 variable characters were parsimony-uninformative, and 279 were parsimony-informative. There were 21 different haplotypes. The best-fit model selected by hLRT in Modeltest 3.5.mac was the HKY+I+G model (Table 1). Figure 2 shows the Neighbour-Joining tree based on the concatenated sequence. All populations within each IRS clustered together, so that each IRS (including the populations Kirindy (West) and Mantadia (East)) built separate terminal clades, supported by high bootstrap values (Fig. 2). The phylogram consists of four major clades, a western, a northwestern, a northern clade, and the clade of *L. mustelinus*. *L. mustelinus *branched off first, followed by the western clade that consisted of IRS 0 and the individuals found in Kirindy (West) (bootstrap values between 93 and 96). The northern clade consisted of IRS IV, V, VI and VII (bootstrap values of 100), and the northwestern clade of IRS I, II and III (bootstrap values of 100). All so far recognized species formed distinct terminal clades with moderate (*L. ankaranensis*, *L. dorsalis*) to large (*L. mustelinus*, *L. ruficaudatus*, *L. aeeclis*) branch lengths. Branch lengths among IRS I, II and III in the northwestern clade were in the same scale as these between *L. ankaranensis *and *L. dorsalis*.

**Figure 3:**
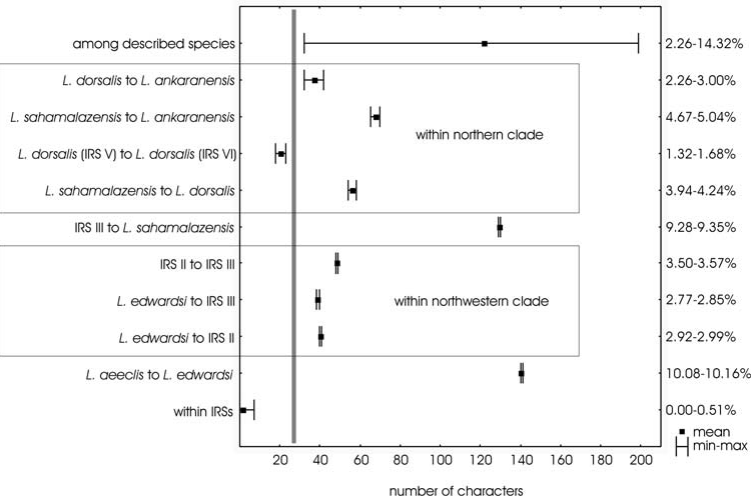
Absolute pairwise distances (minimum-maximum and mean) within IRSs, between neighbouring IRSs/species and among the seven already described species.

The absolute pairwise distances within an IRS ranged from zero to seven characters (Fig. 3). The absolute pairwise distances among IRSs (including Kirindy (West) and Mantadia (East)) varied from 18 to 199 characters. The largest absolute pairwise distance (199 characters) existed between *L. aeeclis *and *L. mustelinus*. The smallest absolute pairwise distance (18 to 23 characters) among IRSs existed between IRS V and IRS VI, both of them were previously supposed to give home to *L. dorsalis*. The relative genetic distance between these two IRSs can be defined as intermediate between the intra-IRS differences (0–7 bp) and the interspecific differences (32–199 bp). This level of differentiation could indicate the presence of two subspecies of *L. dorsalis*. In accordance with the deep phylogenetic splits in the Lepilemur tree among the western, northwestern, and northern clade, absolute pairwise distances were always largest when crossing borders among neighbouring biogeographic zones (*L. aeeclis *to *L. edwardsi *and IRS III to *L. sahamalazensis*, Fig. 3). When examining the absolute pairwise distances among the IRSs within the northwestern clade, they were the same size or even larger than between *L. dorsalis *and *L. ankaranensis*, which are accepted species (Fig. 3).

**Figure 4:**
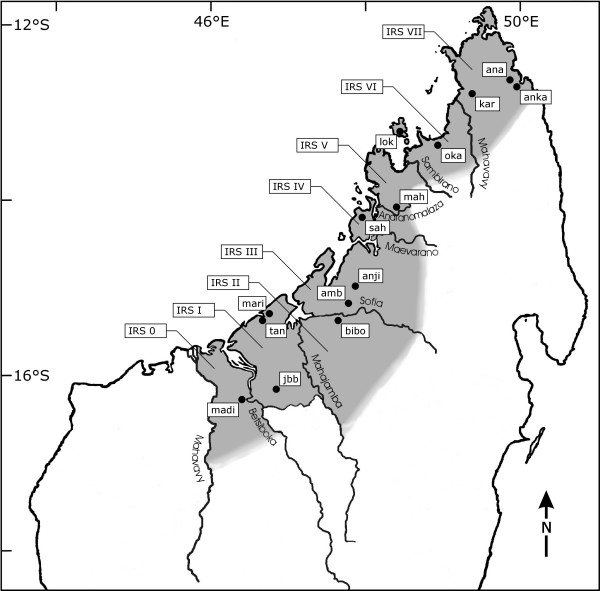
Map of study sites, large rivers and the zonation of the eight Inter-River-Systems (IRSs).

Additional files 1, 2, 3 show the molecular diagnostic sites for each terminal clade in each of the three genes. Recognized species had total number of one (*L. dorsalis*) to 73 (*L. mustelinus*) sites that allowed to identify them unmistakably. The two terminal clades containing the individuals of IRS II and III had a total of eleven and seven diagnostic sites, respectively. The absolute pairwise distances as well as the analysis of the diagnostic sites indicate the presence of two new *Lepilemur *species in northwestern Madagascar, one in IRS II and one in IRS III. Consequently, the geographic range of *L. edwardsi *is much smaller than previously assumed, and limited exclusively to IRS I. By mapping each of these species with respect to their geographical setting (IRS), it can be concluded that all large rivers act as genetic barriers in this genus (Fig. 4).

### Morphometry

The means and standard deviations of all morphometric variables for the nine phylogenetically defined species are provided in Table 2. All variables showed significant differences for species in the ANOVA. Post-hoc tests revealed that ear length and intraorbital distance was significantly different in 15 of the 28 possible pairs of species and thereby the two most distinct variables, followed by snout length (14/28), weight (12/28), head width and lower leg length (10/28), tail circumference (9/28), 3^rd ^toe length (8/28), interorbital distance and hind foot length (6/28) and tail length (4/28). Six variables showed tendencies (0.05 ≤ p < 0.1) in one to two possible pairs of species. The Post-hoc tests revealed significant differences between the northwestern and northern clade and between the northern clade and *L. mustelinus*. Moreover, it could distinguish between all neighbouring species (established and proposed), except between *L. aeeclis *from the western clade and *L. edwardsi *from the northwestern clade (Table 2).

**Table 2:** Descriptive statistics (mean ± SD) for 10 morphometric variables from each species.

	*L. aeeclis * (n = 5)		*L. edwardsi * (n = 11)			*L*. sp. nov. in IRS II (n = 6)			*L*. sp. nov. in IRS III (n = 8)			*L. sahamalazensis * (n = 7)			*L. dorsalis * (n = 30)			*L. ankaranensis * (n = 26)			*L. mustelinus * (n = 7)		Results of ANOVA		
Variable	Mean	SD	Mean	SD		Mean	SD		Mean	SD		Mean	SD		Mean	SD		Mean	SD		Mean	SD	df	F	p

Ear length [mm]	30.72	0.83	31.75	1.93		33.60	0.99		33.41	2.10	**	26.73	1.79		25.59	1.52	**	28.79	1.71	**	31.93	3.11	7	37.081	0.000
Head width [mm]	36.38	0.83	34.93	1.36	*	37.88	2.24		37.61	2.29	**	34.00	1.37		34.69	1.72		34.12	1.97	**	38.36	2.33	7	9.105	0.000
Snout length [mm]	15.50	1.13	17.52	2.25	**	21.00	0.83		19.34	1.45	**	15.10	1.05		14.74	1.69		13.97	1.67	**	18.66	2.14	7	23.894	0.000
Intraorbital distance [mm]	13.60	0.65	14.51	0.79		15.40	1.04		15.29	1.12	**	13.31	0.96	**	12.08	0.89		12.34	0.76		12.79	1.17	7	25.088	0.000
Interorbital distance [mm]	36.98	0.80	37.80	1.33		36.05	1.92		36.88	0.82		34.96	1.39	**	36.82	1.45	**	35.48	1.13	**	38.41	1.77	7	7.470	0.000
Lower leg length [mm]	96.30	1.37	96.22	5.37		101.37	3.63		105.44	2.91		99.27	1.40		98.05	5.88		99.74	3.69	**	113.30	3.94	7	12.874	0.000
Hind foot length [mm]	48.14	2.98	51.96	2.68		50.23	1.43		51.76	3.45		49.00	2.01		49.83	2.31		48.90	2.51	**	54.94	1.45	7	6.953	0.000
3^rd ^toe length [mm]	21.16	0.90	22.24	1.47		22.37	1.62		23.75	1.60		21.70	0.82		20.61	2.38		20.80	1.13	**	25.76	2.91	7	9.127	0.000
Tail length [mm]	260.00	16.58	279.73	14.64	**	253.00	13.58	**	280.63	15.24		257.57	11.87		263.40	15.53		267.88	17.73		252.57	16.16	7	4.006	0.001
Tail circumference [mm]	34.80	0.84	35.73	3.26		40.33	3.27	*	35.25	3.58		33.14	4.38		34.40	2.19		34.42	1.60	**	42.43	6.83	7	9.046	0.000
Body mass [g]	795.20	80.79	934.73	109.06		938.50	116.15		939.50	96.97	**	673.57	120.13		713.07	93.24		706.31	61.71	**	964.57	96.27	7	20.680	0.000

Two asterisks indicate significant differences (p≤0.05) and one asterisk indicate a statistical trend (0.05≤p<0.1) between the neighbouring species/columns.

The discriminant function analysis used five variables for model calculation, ear length, snout length, lower leg length, interorbital distance and intraorbital distance. Two functions were computed explaining a significant part of the morphometric variability between the six established and two proposed species (Wilk's λ = 0.006; F_(35,120) _= 8.355; p < 0.000). Table 3 shows the classification matrix, with correct classification in 82.5% of the cross-validated cases. The differences between the classification accuracy of each species ranged from 60% to 100%. The individuals of *L. aeeclis*, *L*. sp. nova in IRS III and *L. mustelinus *were correctly classified in 100% of the cases. The individuals of *L*. sp. nova in IRS II and *L. ankaranensis *were correctly classified in 80%, and the individuals of *L. edwardsi*, *L. sahamalazensis *and *L. dorsalis *had the smallest percentage of correct classifications (60%). All misclassifications occurred within each major clade, indicating again cryptic speciation within the genus *Lepilemur*.

**Table 3:** Classification matrix of the discriminant function analysis.

	% correct	*L. aeeclis*	*L. edwardsi*	*L*. sp. nov. in IRS II	*L*. sp. nov. in IRS III	*L. sahamalazensis*	*L. dorsalis*	*L. ankaranensis*	*L. mustelinus*
*L. aeeclis*	100.0	5	0	0	0	0	0	0	0
*L. edwardsi*	80.0	1	4	0	0	0	0	0	0
*L*. sp. nov. in IRS II	80.0	0	0	4	1	0	0	0	0
*L*. sp. nov. in IRS III	100.0	0	0	0	5	0	0	0	0
*L. sahamalazensis*	60.0	0	0	0	0	3	1	1	0
*L. dorsalis*	60.0	0	0	0	0	0	3	1	1
*L. ankaranensis*	80.0	0	0	0	0	0	1	4	0
*L. mustelinus*	100.0	0	0	0	0	0	0	0	5
total	82.5	6	4	4	6	3	5	6	6

## Discussion

### Revised phylogeny of the genus Lepilemur

Molecular methods, such as DNA sequencing provide powerful tools to understand diversity and phylogeny [7,17,23-30]. This could be confirmed by our study in sportive lemurs. The phylogenetic trees distinguished all previously described species. Moreover, it provided evidence for two previously unknown species in northwestern Madagascar. The absolute pairwise distances between all species were in the range of those observed in other lemur genera such as *Mirza *[31], *Microcebus *[31-33], *Hapalemur *[34,35] and *Propithecus *[7,36]. The two new taxa occurred in a single IRS (II and III) each. Their phylogenetic position in the tree, the genetic distances and the number of diagnostic sites, suggest a separation at the species level. Similar conclusions were drawn with comparable approaches in other taxa [17,20,27,31,33].

Besides the molecular evidence, the discriminant function analysis of the morphometric data further supported the species status of the two new *Lepilemur *taxa (IRS II and III) in northwestern Madagascar. Between 80% (IRS II) and 100% (IRS III) of the animals were correctly classified into their IRS of origin. The ANOVA of the morphometric data detected significant differences between the northwestern and northern clade and between the northern clade and *L. mustelinus*. Moreover, it could differentiate between the neighbouring species (established and proposed) within the northwestern and northern clade, but it could not distinguish between *L. edwardsi *and *L. aeeclis *that are geographically separated by the river Betsiboka. The differentiation between the northwestern and northern clade, and between the northern clade and *L. mustelinus *is stronger than between the species within these major clades. Although the lack of differentiation between the western and northwestern clade may also partly be due to a sample size effect, it may also suggest cryptic speciation events in the genus *Lepilemur *not only within major clades as it is known in other taxa [37-39], but also between major clades.

The revised phylogeny of the genus *Lepilemur *is based on the combination of molecular differences (genetic distances and diagnostic sites) and morphometric traits. Diagnostic sites are also routinely used in DNA barcoding, which is becoming an increasingly important tool in species identification [40]. Although DNA barcoding requires a large and nearly complete database of sequences to which individuals can be compared [41,42], the diagnostic sites we identified can be seen as first step towards such a database in *Lepilemur*.

### Description of two new species

#### *Lepilemur otto *sp. nov

##### Holotype

**Figure 5:**
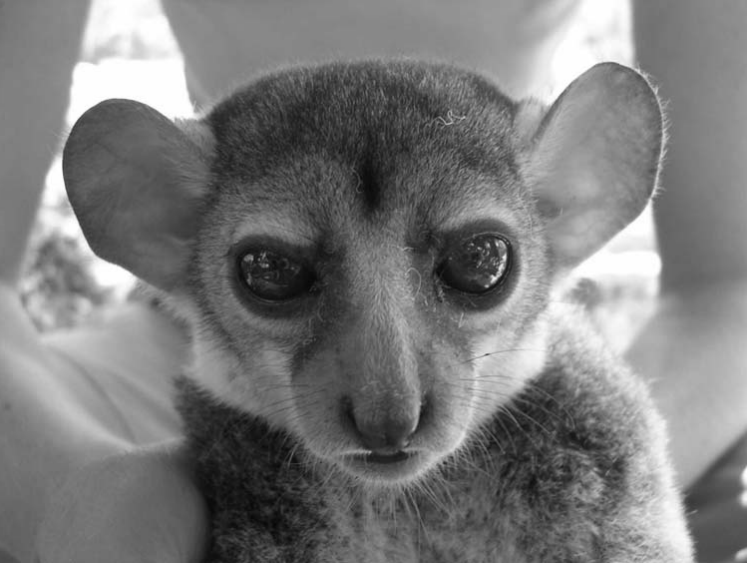
*Lepilemur otto*, portrait of individual 02y04bibo (photograph by M. Craul).

**Figure 6:**
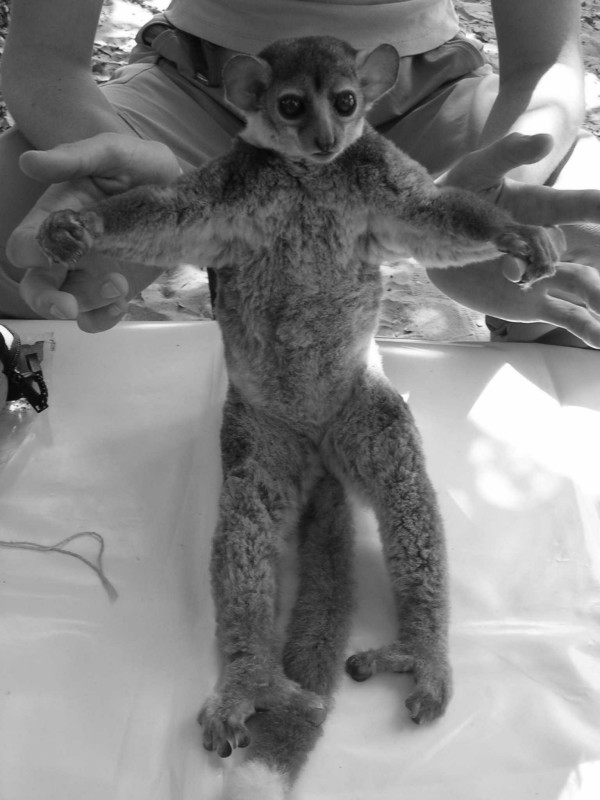
*Lepilemur otto*, body of individual 02y04bibo (photograph by M. Craul).

**Figure 7:**
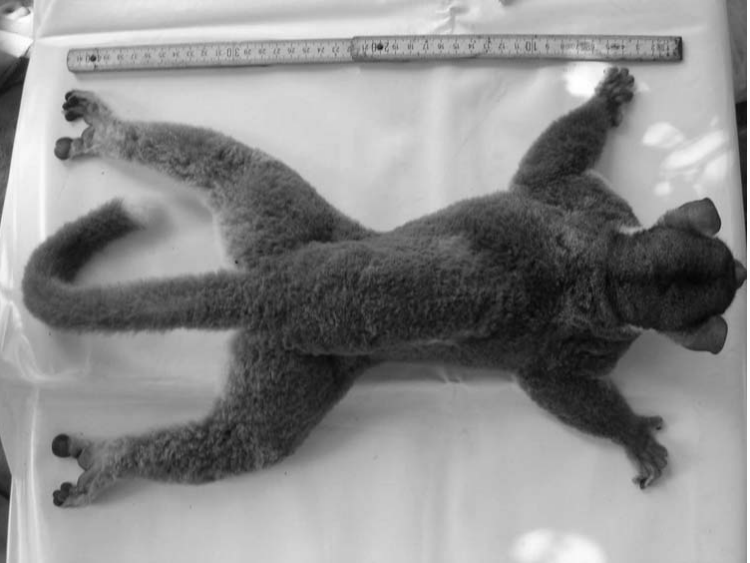
*Lepilemur otto*, back of individual 02y04bibo (photograph by M. Craul).

Individual 02y04bibo, adult male captured in Ambodimahabibo on 1st August 2004 by M. Craul (Fig. 5, 6, 7).

##### Material

Tissue and hair samples, morphometric measurements as well as photographs of 02y04bibo are stored at the Institute of Zoology of the University of Veterinary Medicine Hannover, Hannover, Germany.

##### Type locality

Madagascar: Province de Mahajanga, Ambodimahabibo (15°29'54,2"S, 47°28'47,2"E).

##### Paratype

Individuals 01y04bibo, 03y04bibo and 04y04bibo were captured in Ambodimahabibo by M. Craul in 2004. Tissue and hair samples, morphometric measurements as well as photographs of each paratype are stored at the Institute of Zoology of the University of Veterinary Medicine Hannover, Hannover, Germany.

##### Description

The dorsal pelage, including shoulders and the upper and lower arms, is predominantly grey-brown. A dark diffuse line runs from the middle of the upper skull down the spine, ending in the middle or at the lower part of the back, but is never present on the tail. The ventral pelage is generally grey to creamy. The coloration of the tail is grey-brown to deep brown, sometimes with a white tail tip. The face and forehead are essentially grey.

##### Diagnosis

The sequenced mtDNA of *Lepilemur otto *has eleven diagnostic sites, eight in the ND4 (positions 42 = G, 57 = T, 123 = G, 255 = A, 306 = C, 630 = A, 631 = T, 632 = :C; see additional file 2: Diagnostic sites of the ND4 region for each terminal clade.), and three in the D-loop (positions 20 = C, 22 = A, 23 = T; see additional file 3: Diagnostic sites of the D-loop region for each terminal clade.). *L. otto *differs from its closest relative, *L. edwardsi*, in 2.92–2.99% and from its sister taxon *L. manasamody *in 3.50–3.57% in the sequenced mtDNA, respectively. The few morphometric data, which are available at the moment indicate that *L. otto *has a significant longer snout than the neighbouring species south of the Mahajamba River, *L. edwardsi*. The tail is significant short compared to the neighbouring species north of the Sofia River, *L. manasamody *and to *L. edwardsi*. *L. otto *shows a tendency to have a wider head than *L. edwardsi *and a bigger tail circumference than *L. manasamody*.

##### Distribution

The known distribution range of *Lepilemur otto *is so far limited to the sample site of Ambodimahabibo. This site is situated in the IRS II, which is limited by the Mahajamba River in the west and the Sofia River in the north. Intensive surveys are now required in this vastly deforested area to obtain additional information about the location and viability of other remaining populations, so that conservation measures can be proposed.

##### Etymology

The name *Lepilemur otto *was chosen to acknowledge the donation of Dr. Michael Otto for the purpose of research and conservation of Malagasy lemurs.

##### Vernacular name

Otto's sportive lemur or Lépilemur de Otto.

#### *Lepilemur manasamody *sp. nov

##### Holotype

**Figure 8:**
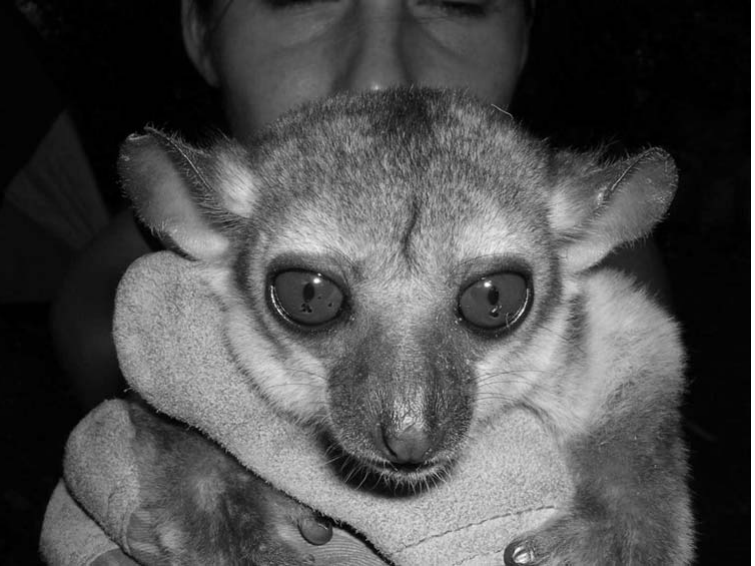
*Lepilemur manasamody*, portrait of individual 16y03amb (photograph by M. Craul).

**Figure 9:**
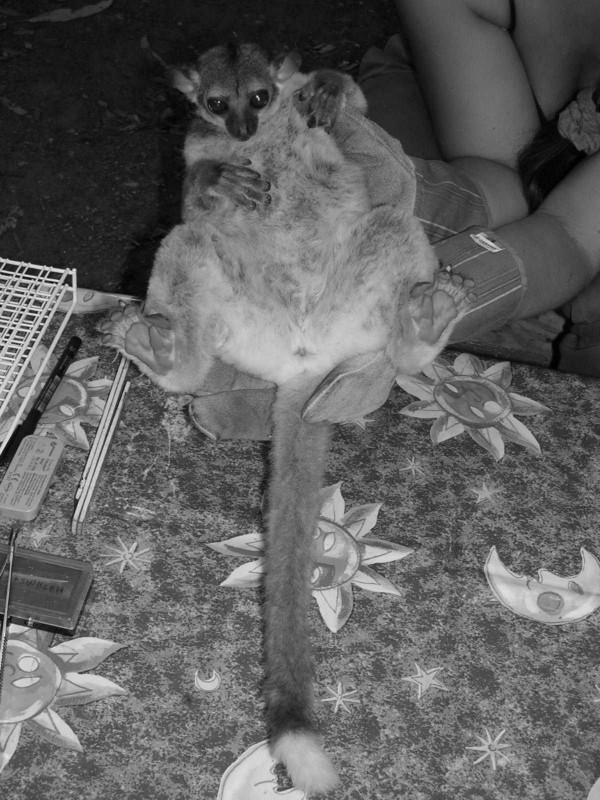
*Lepilemur manasamody*, body of individual 16y03amb (photograph by M. Craul).

**Figure 10:**
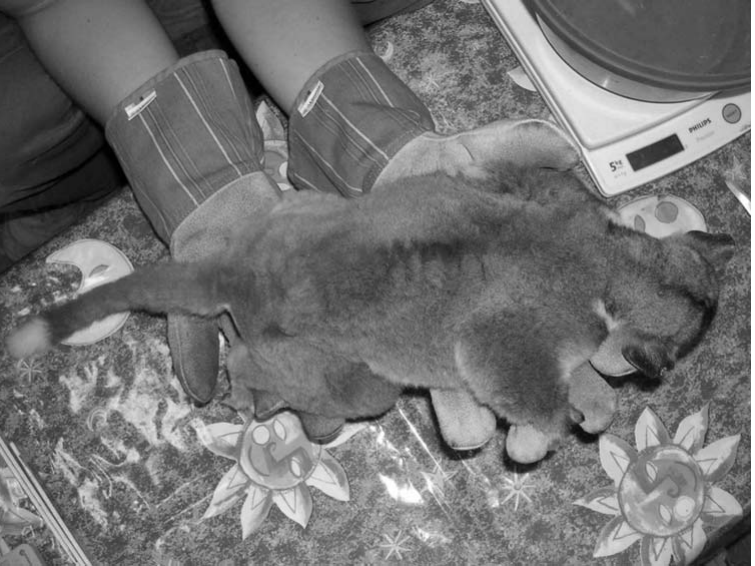
*Lepilemur manasamody*, back of individual 16y03amb (photograph by M. Craul).

Individual 16y03amb, adult female captured in Ambongabe on 20th September 2003 by M. Craul (Fig. 8, 9, 10).

##### Material

Tissue and hair samples, morphometric measurements as well as photographs of 16y03amb are stored at the Institute of Zoology of the University of Veterinary Medicine Hannover, Hannover, Germany.

##### Type locality

Madagascar: Province de Mahajanga, Ambongabe (15°19'38.3"S, 46°40'44.4"E) and Anjiamangirana I (15°09'24.6"S, 47°44'06.2"E).

##### Paratype

Individuals 14y03amb and 15y03amb were captured in Ambongabe and individuals 07y03anji, 08y03anji and 09y03anji in Anjiamangirana I by M. Craul in 2003. Tissue and hair samples, morphometric measurements as well as photographs of all paratypes are stored at the Institute of Zoology of the University of Veterinary Medicine Hannover, Hannover, Germany.

##### Description

The dorsal pelage is predominantly grey-brown, including shoulders, the upper and lower arms. The ventral pelage is generally grey to creamy. The face and forehead are essentially grey. From the middle of the upper skull, a dark diffuse line runs down the spine, ending in the middle of lower part of the back. This line is never present on the tail. The tail is grey-brown to deep brown, sometimes with a white tail tip.

##### Diagnosis

The sequenced mtDNA of *Lepilemur manasamody *has seven diagnostic sites, two of them in the Cytochrome B (positions 86 = G, 140 = G; see additional file 1: Diagnostic sites of the Cytochrome B region for each terminal clade.), three in the ND4 (positions 171 = T, 201 = G, 333 = A; see additional file 2: Diagnostic sites of the ND4 region for each terminal clade.), and two in the D-loop (positions 75 = G, 156 = G; see additional file 3: Diagnostic sites of the D-loop region for each terminal clade.). *L. manasamody *differs from its sister taxa *L. otto *in 3.50–3.57% and from *L. edwardsi *in 2.77–2.92% in the sequenced mtDNA, respectively. The few morphometric data, which are available at the moment, indicate that *L. manasamody *has a significantly longer tail than *L. otto*. *L. manasamody *has significantly longer ears and a longer snout, a significantly wider head and bigger intraorbital distance and is heavier than the neighbouring species to the north, *L. sahamalazensis*. It also shows a tendency to have a smaller tail circumference than *L. otto*.

##### Distribution

The known distribution range of *Lepilemur manasamody *is so far limited to the sample sites of Ambongabe and Anjiamangirana I. Both sites are situated in the IRS III, which is limited by the Sofia River in the south and the Maevarano River in the north. Intensive surveys are now required to obtain additional information about the location and viability of the remaining populations, so that conservation measures can be proposed.

##### Etymology

The name *Lepilemur manasamody *was chosen after the forest region Manasamody, west of Anjiamangirana I between the Sofia and Maevarano River.

##### Vernacular name

Manasamody sportive lemur or Lépilemur de Manasamody.

## Conclusion

Our results showed that all species, except for *L. mustelinus *from the East, grouped in three major clades (western, northwestern and northern). Taking into account the species diversity within each major clade, however, we can define seven biogeographic zones in northern and northwestern Madagascar. When compared to the predictions derived from the "Martin model" and the "Wilmé model", we find several inconsistencies to our data.

The "Martin model" defined four biogeographic zones from western to northern Madagascar (W1, NW, X and N). They corresponded well to the three deep phylogenetic splits, that gave rise to the western, northwestern and northern clade in our study. However, the species diversity within each major clade could not be explained by this model.

The "Wilmé model" defined six biogeographic zones from western to northern Madagascar (numbers 8, 9, 10, 11, 12 and 1). This model may also explain the deep splits between the three major clades, but it proposed two more splits. One between IRS V and VI, divided by the Sambirano River. This split could be confirmed by our study, although it seems not to be a species barrier for sportive lemurs. The level of absolute pairwise distances is intermediate and may rather suggest a variation on a subspecies level. Very recently however, Rabarivola et al. (2006) proposed species status for the individuals in IRS V based on cytogenetics [18]. They collected samples in IRS V at a locality further north of Mahilaka and the number of chromosomes differed between individuals from IRS V (2N = 24) and the neighbouring *Lepilemur sahamalazensis *(2N = 26) and *Lepilemur dorsalis *(2N = 26). The second additional split indicated by the "Wilmé model" is that between zone 12 and 1. This split could not be confirmed by our study, since all individuals found in IRS VII (corresponding to Wilmé's zones 12 and 1) clustered together and belonged to the species *L. ankaranensis*. One major discrepancy exists between our findings and the "Wilmé model". The "Wilmé model" predicts one centre of endemism in northwestern Madagascar (zone 9), which should correspond to one *Lepilemur *species in that area. Our study provided evidence, however, for three species of sportive lemurs between the Betsiboka and Maevarano River, each restricted to one of the three IRSs. Thus, we showed that each IRS is represented as a separate terminal clade in the phylogenetic trees, building distinct phylogenetic units. At least six of the seven large rivers act as species barriers for *Lepilemur*. Therefore, we propose a new model, the "large river model" to explain the biogeography of this larger-sized nocturnal lemur genus. Large rivers acted as insurmountable barriers for gene flow, leading to cryptic speciation within larger biogeographic units. Except for IRSs V and VI, the genetic distances among all IRSs reach species level.

The deep splits between the major clades may indicate initial colonization events, with the Betsiboka and Maevarano River playing a major role in long-term and continuous isolation of western, northwestern and northern Madagascar. The splits within each major clade however, indicate younger cryptic speciation events. Populations, initially belonging to one founder species, entered the IRSs I-III and VI-VII respectively, and were subsequently separated from each other by the rivers Mahajamba and Sofia, and the Andranomalaza, respectively. Quaternary paleoclimatic variation may have played another important role in shaping biogeography and speciation events on Madagascar. The climate during periods of glaciation was cooler and drier than today [8,43,44]. Rivers with year-round water course could have acted as retreats/refugia in times of aridification. All seven large rivers in northwestern and northern Madagascar should have belonged to this category, since the genetic isolation of the IRSs would otherwise not have persisted over time and signs of repeated introgression should be detectable. Subsequent recolonization of the IRSs should thereby have originated from small and isolated refugia, which further promoted genetic differentiation between the IRSs.

In conclusion, we presented evidence for an unexpected species diversity of sportive lemurs in northwestern and northern Madagascar. Current biogeographic models were not sufficient to explain the underlying processes of speciation. We therefore suggest a new model of biogeographical zonation, the "large river model". In this model, biogeographic zones are separated and maintained over time by all large rivers with permanent water bodies that may have provided retreat zones during periods of aridification and may have harboured founder populations for subsequent recolonization. The importance of large rivers as biogeographic barriers was previously emphazized for mouse lemurs [3], but also for neotropical primates [45,46]. Further studies are now needed to test the relevance of this model for other terrestrial taxa, such as the insectivores, rodents, or other lemurs.

## Methods

### Fieldwork

A total of 157 *Lepilemur *individuals were captured at 14 different localities along a 560 km transect from western to northern Madagascar (Fig. 4, Table 4). This region is divided by eight large rivers (over 50 km wide 20 km inlands) into eight Inter-River-Systems (IRS 0 to IRS VII, Fig. 4). Six localities were sampled by Mathias Craul (MC) and eight localities were sampled by Solofo Rasoloharijaona (SR) and Blanchard Randrianambinina (BR). At each site we performed daily and nightly surveys to capture the animals. At daytime we used a net to capture the animals out of their sleeping holes and briefly anesthetised them with Ketasel-5 (Selectavet). At night time we anesthetised the animals using a blowpipe (TELINJECT B22T) with Ketasel-5 (Selectavet). Each captured sportive lemur was then characterised with regard to sex, skin colour, reproductive status (testis size or form of vulva), 13 external morphometric measures (ear length, ear width, head length, head width, snout length, interorbital distance, intraorbital distance, lower leg length, hind foot length, 3^rd ^toe length, body length, tail length, tail circumference) and body mass [10,47]). In addition, a small biopsy from one or both pinnae was taken as tissue samples. Tissue samples were stored in Queen's lysis buffer [48] for later DNA extraction and genetic analyses.

**Table 4:** Details of study sites.

Locality	Abbreviation	Coordinates	Origin
Madirovalo	madi	16°22'45.6"S, 46°29'01.9"E	IRS 0
Ampijoroa	jbb	16°17'S, 46°48'E	IRS I
Mariarano	mari	15°28'50.3"S, 46°41'19.0"E	IRS I
Tananvaovao	tan	15°28'15.5"S, 46°39'59.4"E	IRS I
Ambodimahabibo	bibo	15°29'54.2"S, 47°28'47.2"E	IRS II
Ambongabe	amb	15°19'38.3"S, 47°40'44.4"E	IRS III
Anjiamangirana I	anji	15°09'24.6"S, 47°44'06.2"E	IRS III
Ankarafa	sah	14°22'47.8"S, 47°45'26.3"E	IRS IV
Mahilaka	mah	14°17'12.0"S, 48°12'12.0"E	IRS V
Lokobe	lok	13°23'23.9"S, 48°20'31.0"E	IRS VI
Manehoka	oka	13°25'49.0"S, 48°47'51.0"E	IRS VI
Ankavana	anka	12°46'55.7"S, 49°22'27.4"E	IRS VII
Ankarana	kar	12°58'05.0"S, 49°08'18.0"E	IRS VII
Analabe	ana	12°45'13.8"S, 49°30'03.9"E	IRS VII
Kirindy	kir	20°03'S, 44°37'E	West
Mantadia	man	18°47'S, 48°25'E	East

### Molecular methods and analyses

DNA from the tissue of 37 individuals was isolated with the DNeasy Tissue Kit (Qiagen), or extracted using a standard proteinase K digestion followed by a Phenol/Chloroform protocol [49] and stored at -20°C. In addition, we analysed the DNA of two individuals of *Lepilemur ruficaudatus *(Kirindy forest, western Madagascar) provided by Yves Rumpler and of four individuals from Mantadia (eastern Madagascar) sampled previously by SR and BR. We sequenced the mitochondrial genes D-loop, cytochrome B and NADH-dehydrogenase subunit 4 (ND4), because reference sequences from all eleven recognized species were available for these particular markers. The complete D-loop was amplified with the oligonucleotide primers DLp-1.5: 5'-GCA CCC AAA GCT GAR RTT CTA-3' and DLp-5: 5'-CCA TCG WGA TGT CTT ATT TAA GRG GAA-3' [19]. Standard PCRs were carried out in a 25 μl reaction with a final concentration of 1 μM for each primer, 1.5 mM for MgCl_2_, 0.2 mM for each dNTP, 1 × NH_4 _reaction buffer (50 mM Tris-HCl pH 8.8, 16 mM (NH_4_)_2 _SO_4_, 0.1% Tween^® ^20), 1.25 units of Taq DNA polymerase, and 1 μl of DNA. Successful amplifications were obtained using the following protocol: 35 cycles of denaturing at 94°C for 60 seconds, primer annealing at 47°C for 60 seconds and extension at 72°C for 90 seconds. The partial Cytochrome B was amplified with the oligonucleotide primers L14841: 5'-AAA AAG CTT CCA TCC AAC ATC TCA GCA TGA TGA AA-3' and H15149: 5'-AAA CTG CAG CCC CTC AGA ATG ATA TTT GTC CTC A-3' [50]. Standard PCRs were carried out in a 25 μl reaction with a final concentration of 1 μM for each primer, 1.5 mM for MgCl_2_, 0.2 mM for each dNTP, 1 × NH_4 _reaction buffer (50 mM Tris-HCl pH 8.8, 16 mM (NH_4_)_2 _SO_4_), 1.25 units of Taq DNA polymerase, and 1 μl of DNA. Successful amplifications were obtained using the following protocol: 35 cycles of denaturing at 94°C for 60 seconds, primer annealing at 47°C for 60 seconds and extension at 72°C for 90 seconds. The partial NADH-dehydrogenase subunit 4 was amplified with the oligonucleotide primers LepiP1: 5'-TTG ATG TAG TAT GAC TRT TCC-3' and LepiR1: 5'-GCC AAA CCG ATG GCT GCT TCA CAG GCT GCA AG-3' [51]. Standard PCRs were carried out in a 25 μl reaction with a final concentration of 1 μM for each primer, 1.5 mM for MgCl_2_, 0.2 mM for each dNTP, 1 × NH_4 _reaction buffer (50 mM Tris-HCl pH 8.8, 16 mM (NH_4_)_2 _SO_4_), 1.25 units of Taq DNA polymerase, and 1 μl of DNA. Successful amplifications were obtained using the following protocol: 40 cycles of denaturing 95°C for 30 seconds, primer annealing at 60°C for 60 seconds and extension at 72°C for 60 seconds. The PCR products were cleaned with the Invisorb Spin PCRapid Kit (Invitek) or Quick-Clean (Bioline) and checked for successful amplification by running an aliquot on a 1.5% agarose gel, stained with 1.3 × 10^-4 ^mg/ml ethidium bromide. After cleaning the PCR products, cycle sequencing reactions were carried out using DYEnamic™ ET dye terminator kit (Amersham Biosciences) and the primers indicated above. After a second cleaning with ammonium acetate, provided with the DYEnamic™ ET dye terminator kit, the PCR products were sequenced on a MegaBACE™ 1000 DNA Sequencing System (Amersham Biosciences). The respective sequences were deposited in GenBank (Table 5).

**Table 5:** Locality, origin, sample type and GenBank accession number of analysed individuals for genetic studies.

Species	Locality	Abbreviation	Origin	Sample type	D-loop	Cyt B	ND4
*L. ruficaudatus*	Kirindy	kir1	west	DNA	EF686766	EF686723	EF686680
*L. ruficaudatus*	Kirindy	kir2	west	DNA	EF686767	EF686724	EF686681
*L. ruficaudatus*	Kirindy		west	sequence		DQ109013–DQ109015, DQ109017	AF224596
*L. randrianasoli*			west	sequence		AY321456	
*L. randrianasoli*	Andramasay		west	sequence		AY441463, DQ109019, DQ234891–DQ234894	
*L. randrianasoli*	Ambalarano		west	sequence		DQ234890	
*L. aeeclis*	Madirovalo	madi1	IRS 0	tissue	EF686768	EF686725	EF686682
*L. aeeclis*	Madirovalo	madi2	IRS 0	tissue	EF686769	EF686726	EF686683
*L. aeeclis*	Anjamena		IRS 0	sequence			AF224593
*L. aeeclis*	Antafia-Anjahamena		IRS 0	sequence		DQ108999–DQ109003, DQ234899	
*L. edwardsi*	Ampijoroa	jbb1	IRS I	tissue	EF686756	EF686713	EF686670
*L. edwardsi*	Ampijoroa	jbb2	IRS I	tissue	EF686757	EF686714	EF686671
*L. edwardsi*	Mariarano	mari1	IRS I	tissue	EF686760	EF686717	EF686674
*L. edwardsi*	Mariarano	mari2	IRS I	tissue	EF686761	EF686718	EF686675
*L. edwardsi*	Tananvaovao	tan1	IRS I	tissue	EF686758	EF686715	EF686672
*L. edwardsi*	Tananvaovao	tan2	IRS I	tissue	EF686759	EF686716	EF686673
*L. edwardsi*	Ampijoroa		IRS I	sequence		DQ109006	AF224595
*L. edwardsi*	Andofombombe		IRS I	sequence		DQ109004, DQ109005, DQ234888	
*L*. sp. nov. IRS II	Ambodimahabibo	bibo1	IRS II	tissue	EF686762	EF686719	EF686676
*L*. sp. nov. IRS II	Ambodimahabibo	bibo2	IRS II	tissue	EF686763	EF686720	EF686677
*L*. sp. nov. IRS II	Ambodimahabibo	bibo3	IRS II	tissue	EF686764	EF686721	EF686678
*L*. sp. nov. IRS II	Ambodimahabibo	bibo4	IRS II	tissue	EF686765	EF686722	EF686679
*L*. sp. nov. IRS III	Ambongabe	amb1	IRS III	tissue	EF686753	EF686710	EF686667
*L*. sp. nov. IRS III	Ambongabe	amb2	IRS III	tissue	EF686754	EF686711	EF686668
*L*. sp. nov. IRS III	Anjiamangirana I	anji1	IRS III	tissue	EF686755	EF686712	EF686669
*L. sahamalazensis*	Ankarafa	sah1	IRS IV	tissue	EF686750	EF686707	EF686664
*L. sahamalazensis*	Ankarafa	sah2	IRS IV	tissue	EF686751	EF686708	EF686665
*L. sahamalazensis*	Ankarafa	sah3	IRS IV	tissue	EF686752	EF686709	EF686666
*L. sahamalazensis*	Sahamalaza		IRS IV	sequence		DQ108990–DQ108992, DQ234882, DQ234883	
*L. dorsalis*	Mahilaka	mah1	IRS V	tissue	EF686746	EF686703	EF686660
*L. dorsalis*	Mahilaka	mah2	IRS V	tissue	EF686747	EF686704	EF686661
*L. dorsalis*	Mahilaka	mah3	IRS V	tissue	EF686748	EF686705	EF686662
*L. dorsalis*	Ambanja		IRS V	sequence		DQ108995–DQ108997, DQ234886, DQ234887	
*L. dorsalis*	Mahilaka	mah4	IRS V	tissue	EF686749	EF686706	EF686663
*L. dorsalis*	Lokobe	lok1	IRS VI	tissue	EF686740	EF686697	EF686654
*L. dorsalis*	Lokobe	lok3	IRS VI	tissue	EF686741	EF686698	EF686655
*L. dorsalis*	Manehoka	oka1	IRS VI	tissue	EF686744	EF686701	EF686658
*L. dorsalis*	Manehoka	oka2	IRS VI	tissue	EF686742	EF686699	EF686656
*L. dorsalis*	Manehoka	oka3	IRS VI	tissue	EF686743	EF686700	EF686657
*L. dorsalis*	Manehoka	oka4	IRS VI	tissue	EF686745	EF686702	EF686659
*L. dorsalis*	Nosy Be		IRS VI	sequence		AY441464, DQ108993, DQ108994, DQ108998, DQ234885	
*L. ankaranensis*	Ankavana	anka1	IRS VII	tissue	EF686735	EF686692	EF686649
*L. ankaranensis*	Ankavana	anka2	IRS VII	tissue	EF686734	EF686691	EF686648
*L. ankaranensis*	Ankavana	anka3	IRS VII	tissue	EF686736	EF686693	EF686650
*L. ankaranensis*	Ankarana	kar1	IRS VII	tissue	EF686731	EF686688	EF686645
*L. ankaranensis*	Ankarana	kar2	IRS VII	tissue	EF686732	EF686689	EF686646
*L. ankaranensis*	Ankarana	kar3	IRS VII	tissue	EF686733	EF686690	EF686647
*L. ankaranensis*	Analabe	ana1	IRS VII	tissue	EF686737	EF686694	EF686651
*L. ankaranensis*	Analabe	ana2	IRS VII	tissue	EF686738	EF686695	EF686652
*L. ankaranensis*	Analabe	ana3	IRS VII	tissue	EF686739	EF686696	EF686653
*L. ankaranensis*	Ankarana		IRS VII	sequence		DQ109028–DQ109032	AF304597
*L. ankaranensis*	Analamera		IRS VII	sequence		DQ109022–DQ109024, DQ234884	
*L. ankaranensis*	Andrafiamena		IRS VII	sequence		DQ109025, DQ109027, DQ234881	
*L. septentrionalis*	Sahafary		IRS VII	sequence	AJ304651	DQ109020, DQ109021, DQ234900	
*L. mustelinus*	Mantadia	man1	east	tissue	EF686727	EF686684	EF686641
*L. mustelinus*	Mantadia	man2	east	tissue	EF686728	EF686685	EF686642
*L. mustelinus*	Mantadia	man3	east	tissue	EF686729	EF686686	EF686643
*L. mustelinus*	Mantadia	man4	east	tissue	EF686730	EF686687	EF686644
*L. mustelinus*	Behasina		east	sequence		DQ109033	
*L. mustelinus*	near Mantadia		east	sequence		DQ109034	
*L. microdon*	Vohiparara		east	sequence		DQ109008	
*L. microdon*	Antarando		east	sequence		DQ109009, DQ109010	
*L. microdon*	Ambatolampy		east	sequence		DQ234889	
*L. leucopus*			south	sequence		DQ109007	
*P. diadema*				sequence	AF354743	AY441452	AF224599

For a comprehensive phylogenetic analysis of the sequence data, we expanded our data set with reference sequences from all eleven recognized species available from GenBank (Table 5). As outgroup for phylogenetic tree reconstructions, we selected *Propithecus diadema*. Sequences were aligned using the program CLUSTALX [52] and checked by eye. Tree reconstructions of each single gene were carried out to phylogenetically classify the sampled individuals within the genus *Lepilemur*. Because of the lack of reference sequences of single individuals for all three genes, further phylogenetic tree reconstructions based on all three genes were performed only with our own data set consisting of 43 sequences. Phylogenetic tree reconstructions were carried out with the maximum-parsimony (MP), neighbour-joining (NJ) and maximum-likelihood (ML) algorithms as implemented in PAUP4.0b10 [53]. Throughout the analyses, all characters were treated as unordered and equally weighted. Gaps were considered as missing data in NJ and ML, but were treated as fifth character in MP analysis. The NJ and ML trees were constructed using the best-fit model selected by the hierarchical likelihood ratio test (hLRT) in Modeltest 3.5.mac [54]. Relative support of internal nodes was provided by bootstrap analyses with 1,000 replications for MP and NJ and 100 replications for ML. Absolute pairwise distances were calculated using PAUP4.0b10 [50] and ARLEQUIN 1.1 to describe the variation among taxa. To determine fixed molecular differences among terminal clades (indicating barriers for gene flow), diagnostic sites for each terminal clade to all others were identified using the program MEGA 3.1 [55].

### Statistical analyses of morphometric data

Quantitative analyses of morphometric data were carried out with two different sample sizes. The ANOVA was conducted with 100 individuals. After removing two variables that differed among researchers (1-way ANOVA, STATISTICA 6.0, Statsoft, Inc.), the 11 remaining variables were tested for normality using the Kolmogorov-Smirnov test (Statistica 6.0, StatSoft, Inc.) at a level of p ≤ 0.05. All were normally distributed. A MANOVA revealed no differences in sex. The variables were then tested for correlation. All variables had an r < 0.75 and were therefore defined as sufficiently independent to be used in a discriminant function analysis [56]. This analysis was limited to five adult individuals per species (established and proposed) in order to equilibrate the samples. The discriminant function analysis tested only for species differences and for differences between the IRSs. A stepwise forward method (statistic: Wilk's λ) with the criteria F_*toenter *_= 3.84 and F_*toremove *_= 2.71 and a tolerance level of p ≤ 0.01 was used to calculate the discriminant function model. The computed discriminant functions were used to classify cases with regard to their group membership. All cases were cross-validated by the "leave-one-out" method, where each case in the analysis is classified by the functions derived from all cases other than that case. The discriminant function analysis was carried out with the program SPSS 13.0 (SPSS, Inc.).

## Footnote

During the review process of this paper, Louis Jr. et al. (2006) described a new sportive lemur species in IRS III [57]. It was named *Lepilemur grewcocki*. This might be a synonym to *L. manasamody*, as our sampling sites of this species were in the same IRS. However, a joint phylogenetic analysis is still needed to verify the identity of both forms.

## Authors' contributions

MC participated in the design of the study, conducted part of the field work and all lab work, performed the comparative genetic and morphometric analyses and wrote the MS.

EZ initiated, financed and designed the study. She organized and conceptualized the field work, supervised data analyses and critically revised the MS several times and approved its final version.

SR conducted part of the field work including capturing, measuring and sampling *L. ankaranensis*, *L. dorsalis*, *L. mustelinus *and *L. aeeclis*.

BR conducted part of the field work including capturing, measuring and sampling *L. ankaranensis*, *L. dorsalis*, *L. mustelinus *and *L. aeeclis*.

UR designed the study. She organized and conceptualized the field work, supervised data analyses and critically revised the MS several times and approved its final version.

## Supplementary Material

Additional file 1 **Diagnostic sites of the Cytochrome B region for each terminal clade. **Dashes (-) indicate deletions. Points (.) indicate identical bases. Click here for file

Additional file 2 **Diagnostic sites of the ND4 region for each terminal clade. **Dashes (-) indicate deletions. Points (.) indicate identical bases. Click here for file

Additional file 3 **Diagnostic sites of the D-loop region for each terminal clade. **Dashes (-) indicate deletions. Points (.) indicate identical bases. Click here for file

## References

[B1] Martin RD (1990). Primate Origins and Evolution: A Phylogenetic Reconstruction.

[B2] Mittermeier RA, Konstant WR, Hawkins F, Louis EE, Langrand O, Ratsimbazafy J, Rasoloarion R, Ganzhorn JU, Rajaobelina S, Tattersall I, Meyers DM (2006). Lemurs of Madagascar.

[B3] Olivieri G, Zimmermann E, Randrianambinina B, Rasoloharijaona S, Rakotondravony D, Guschanski K, Radespiel U (2006). The ever-increasing diversity in mouse lemurs: three new species in north and northwestern Madagascar. Mol Phylogenet Evol.

[B4] Ganzhorn JU, Langrand O, Wright PC, O'Connor S, Rakotosamimanana B, Feistner ATC, Rumpler Y (1996). The state of lemur conservation in Madagascar. Prim Conserv.

[B5] Martin RD (1972). Adaptive radiation and behaviour of the Malagasy lemurs. Philos Trans R Soc Lond B Biol Sci.

[B6] Martin RD, Alterman L, Doyle GA, Izard MK (1995). Prosimians: from obscurity to extinction?. Creatures of the dark: the nocturnal prosimians.

[B7] Pastorini J, Thalmann U, Martin RD (2003). A molecular approach to comparative phylogeography of extant Malagasy lemurs. Proc Natl Acad Sci USA.

[B8] Wilmé L, Goodman S, Ganzhorn J (2006). Biogeographic evolution of Madagascar's microendemic biota. Science.

[B9] Rasoloharijaona S, Rakotosamimanana B, Randrianambinina B, Zimmermann E (2003). Pair-specific usage of sleeping sites and their implications for social organization in a nocturnal Malagasy primate, the Milne Edwards' sportive lemur (*Lepilemur edwardsi*). Am J Phys Anthropol.

[B10] Rasoloharijaona S, Randrianambinina B, Braune P, Zimmermann E (2006). Loud calling, spacing, and cohesiveness in a nocturnal primate, the Milne Edwards' sportive lemur (*Lepilemur edwardsi*). Am J Phys Anthropol.

[B11] Thalmann U, Ganzhorn J, Goodmann SM, Benstead JP (2003). *Lepilemur*, Sportive Lemur. The Natural History of Madagascar.

[B12] Petit G (1933). Le genre Lepidolemur et sa répartition géographique. C R Soc Biogéogr.

[B13] Petter JJ, Petter-Rousseaux A (1960). Remarque sur la systématique du genre *Lepilemur*. Mammalia.

[B14] Petter JJ, Albignac R, Rumpler Y (1977). Mammifères lémuriens (Primates prosimiens). Faune de Madagascar.

[B15] Rumpler Y, Albignac R (1978). Chromosomes studies of the Lepilemur, an endemic Malagasy genus of lemurs: Contribution of the cytogenetics to their taxonomy. J Hum Evol.

[B16] Tattersall I (1982). The primates of Madagascar.

[B17] Andriaholinirina N, Fausser JL, Roos C, Zinner D, Thalmann U, Rabarivola C, Ravoarimanana I, Ganzhorn JU, Meier B, Hilgartner R, Walter L, Zaramody A, Langer C, Hahn T, Zimmermann E, Radespiel U, Craul M, Tomiuk J, Tattersall I, Rumpler Y (2006). Molecular phylogeny and taxonomic revision of the sportive lemurs (*Lepilemur*, Primates). BMC Evol Biol.

[B18] Rabarivola C, Zaramody A, Fausser JL, Andriaholinirina N, Roos C (2006). Cytogenetic and molecular characteristics of a new species of sportive lemur from Northern Madagascar. Lemur News.

[B19] Wyner YM, Amato G, Desalle R (1999). Captive breeding, reintroduction, and the conservation genetics of black and white ruffed lemurs, *Varecia variegata variegata*. Mol Ecol.

[B20] Ravaoarimanana IB, Tiedemann R, Montagnon D, Rumpler Y (2004). Molecular and cytogenetic evidence for cryptic speciation within a rare endemic Malagasy lemur, the Northern Sportive Lemur (*Lepilemur septentrionalis*). Mol Phylogenet Evol.

[B21] Cracraft J (1983). Species concepts and speciation analysis. Current Ornithology.

[B22] Davis JI, Nixon KC (1992). Populations, genetic variation and the delimitation of phylogenetic species. Syst Biol.

[B23] Yoder AD, Olson LE, Hanley C, Heckman KL, Rasoloarison R, Russell AL, Ranivo J, Soarimalala V, Karanth KP, Raselimanana AP, Goodman SM (2005). A multidimensional approach for detecting species patterns in Malagasy vertebrates. Proc Natl Acad Sci USA.

[B24] Vences M, Vieites DR, Glaw F, Brinkmann H, Kosuch J, Veith M, Meyer A (2003). Multiple overseas dispersal in amphibians. Proc Biol Sci.

[B25] Yoder AD, Burns MM, Zehr S, Delefosse T, Veron G, Goodman SM, Flynn JJ (2003). Single origin of Malagasy carnivora from an African ancestor. Nature.

[B26] Jansa SA, Barker FK, Heaney LR (2006). The pattern and timing of diversification of Philippine endemic rodents: evidence from mitochondrial and nuclear gene sequences. Syst Biol.

[B27] Asher RJ, Hofreiter M (2006). Tenrec phylogeny and the noninvasive extraction of nuclear DNA. Syst Biol.

[B28] Farias IP, Orti G, Sampaio I, Schneider H, Meyer A (1999). Mitochondrial DNA phylogeny of the family Cichlidae: monophyly and fast molecular evolution of the neotropical assemblage. J Mol Evol.

[B29] Nagy ZT, Joger U, Wink M, Glaw F, Vences M (2003). Multiple colonization of Madagascar and Socotra by colubrid snakes: evidence from nuclear and mitochondrial gene phylogenies. Proc Biol Sci.

[B30] Cooper A, Lalueza-Fox C, Anderson S, Rambaut A, Austin J, Ward R (2001). Complete mitochondrial genome sequences of two extinct moas clarify ratite evolution. Nature.

[B31] Kappeler PM, Rasoloarison RM, Razafimanantsoa L, Walter L, Roos C (1870). Morphology, Behaviour and Molecular Evolution of Giant Mouse Lemurs (*Mirza *spp.) Gray, with Description of a new Species. Primate Report.

[B32] Pastorini J, Martin RD, Ehresmann P, Zimmermann E, Forstner MRJ (2001). Molecular phylogeny of the lemur family Cheirogaleidae (Primates) based on mitochondrial DNA sequences. Mol Phylogenet Evol.

[B33] Yoder AD, Rasoloarison RM, Goodman SM, Irwin JA, Atsalis S, Ravosa MJ, Ganzhorn JU (2000). Remarkable species diversity in Malagasy mouse lemurs (primates, Microcebus). Proc Natl Acad Sci USA.

[B34] Fausser JL, Prosper P, Donati G, Ramanamanjato JB, Rumpler Y (2002). Phylogenetic relationships between *Hapalemur *species and subspecies based on mitochondrial DNA sequences. BMC Evol Biol.

[B35] Pastorini J, Forstner MRJ, Martin RD (2002). Phylogenetic relationships among Lemuridae (Primates): evidence from mtDNA. J Hum Evol.

[B36] Mayor MI, Sommer JA, Houck ML, Zaonarivelo JR, Wright PC, Ingram C, Engel SR, Louis EE (2004). Specific status of *Propithecus *spp. Int J Primatol.

[B37] Camargo A, de Sá RO, Heyer WR (2006). Phylogenetic analyses of mtDNA sequences reveal three cryptic lineages in the widespread neotropical frog *Leptodactylus fuscus *(Schneider, 1799) (Anura, Leptodactylidae). Biol J Linnean Soc.

[B38] Goetze E (2003). Cryptic speciation on the high seas; global phylogenetics of the copepod family Eucalanidae. Proc Biol Sci.

[B39] Piaggio AJ, Perkins SL (2005). Molecular phylogeny of North American long-eared bats (Vespertilionidae: Corynorhinus); inter- and intraspecific relationships inferred from mitochondrial and nuclear DNA sequences. Mol Phylogenet Evol.

[B40] DeSalle R (2006). Species Discovery versus Species Identification in DNA Barcoding Efforts: Response to Rubinoff. Cons Biol.

[B41] Moritz C, Cicero C (2004). DNA barcoding: promise and pitfalls. PloS Biol.

[B42] Will KW, Rubinoff D (2004). Myth of the molecule: DNA barcodes for species cannot replace morphology for identification and classification. Cladistics.

[B43] Haffer J (1969). Speciation in Amazonian Forest Birds. Science.

[B44] de Wit MJ (2003). MADAGASCAR: Heads It's a Continent, Tails It's an Island. Annual Review of Earth and Planetary Sciences.

[B45] Ayres JM, Clutton-Brock TH (1992). River boundaries and species range size in Amazonian primates. American Naturalist.

[B46] Lehman SM (2004). Distribution and Diversity of Primates in Guyana: Species-Area Relationships and Riverine Barriers. Int J Primatol.

[B47] Zimmermann E, Cepok S, Rakotoarison N, Zietemann V, Radespiel U (1998). Sympatric mouse lemurs in north-west Madagascar: a new rufous mouse lemur species (*Microcebus ravelobensis*). Folia Primatol.

[B48] Seutin G, White BN, Boag PT (1991). Preservation of avian blood and tissue samples for DNA analyses. Can J Zool.

[B49] Sambrook J, Fritsch E, Maniatis T (1989). Molecular Cloning: A Laboratory Manual.

[B50] Kocher TD, Thomas WK, Meyer A, Edwards SV, Pääbo S, Villablanca FX, Wilson AC (1989). Dynamics of mitochondrial DNA evolution in animals: Amplification and sequencing with conserved primers. Proc Natl Acad Sci USA.

[B51] Pastorini J (2000). Molecular Systematics of Lemurs. PhD thesis.

[B52] Thompson JD, Gibson TJ, Plewniak F, Jeanmougin F, Higgins DG (1997). The CLUSTAL_X windows interface: flexible strategies for multiple sequence alignment aided by quality analysis tools. Nucleic Acids Res.

[B53] Swofford DL (1999). PAUP*: Phylogenetic analysis using parsimony (*and other methods), version 40 b2.

[B54] Posada D, Crandall KA (1998). MODELTEST: testing the model of DNA substitution. Bioinformatics.

[B55] Kumar S, Tamura K, Nei M (2004). MEGA3: Integrated software for Molecular Evolutionary Genetics Analysis and sequence alignment. Brief Bioinform.

[B56] Braune P, Schmidt S, Zimmermann E (2005). Spacing and group coordination in a nocturnal primate, the golden brown mouse lemur (*Microcebus ravelobensis*): the role of olfactory and acoustic signals. Behav Ecol Sociobiol.

[B57] Louis EE, Engberg SE, Lei R, Geng H, Sommer JA, Randriamampionona R, Randriamanana JC, Zaonarivelo JR, Andriantompohavana R, Randria G, Prosper, Ramaromilanto B, Rakotoarisoa G, Rooney A, Brenneman RA (2006). Molecular and Morphological Analyses of the Sportive Lemurs (Family Megaladapidae: Genus *Lepilemur*) Reveals 11 Previously Unrecognized Species. Special Publications.

